# Effects of Resveratrol Supplementation on Methotrexate Chemotherapy-Induced Bone Loss

**DOI:** 10.3390/nu9030255

**Published:** 2017-03-09

**Authors:** Alice M. C. Lee, Tetyana Shandala, Pei Pei Soo, Yu-Wen Su, Tristan J. King, Ke-Ming Chen, Peter R. Howe, Cory J. Xian

**Affiliations:** 1 Sansom Institute for Health Research, School of Pharmacy and Medical Sciences, University of South Australia, Adelaide SA 5001, Australia; alice.lee@unisa.edu.au (A.M.C.L.); tetyanas@geneworks.com.au (T.S.); soopp001@students.unisa.edu.au (P.P.S.); yu-wen.su@unisa.edu.au (Y.-W.S); tristan.king@unisa.edu.au (T.J.K.); 2 Institute of Orthopaedics, Lanzhou General Hospital, Lanzhou Command of People’s Liberation Army, Lanzhou 730050, China; chenkm@lut.cn; 3 Clinical Nutrition Research Centre, University of Newcastle, Callaghan NSW 2308, Australia; peter.howe@newcastle.edu.au

**Keywords:** resveratrol, cancer chemotherapy, methotrexate, bone loss, bone marrow adiposity, bone growth arrest, growth plate, osteoclasts, osteoblasts, adipocytes

## Abstract

Intensive cancer chemotherapy is known to cause bone defects, which currently lack treatments. This study investigated the effects of polyphenol resveratrol (RES) in preventing bone defects in rats caused by methotrexate (MTX), a commonly used antimetabolite in childhood oncology. Young rats received five daily MTX injections at 0.75 mg/kg/day. RES was orally gavaged daily for seven days prior to, and during, five-day MTX administration. MTX reduced growth plate thickness, primary spongiosa height, trabecular bone volume, increased marrow adipocyte density, and increased mRNA expression of the osteogenic, adipogenic, and osteoclastogenic factors in the tibial bone. RES at 10 mg/kg was found not to affect bone health in normal rats, but to aggravate the bone damage in MTX-treated rats. However, RES supplementation at 1 mg/kg preserved the growth plate, primary spongiosa, bone volume, and lowered the adipocyte density. It maintained expression of genes involved in osteogenesis and decreased expression of adipogenic and osteoclastogenic factors. RES suppressed osteoclast formation ex vivo of bone marrow cells from the treated rats. These data suggest that MTX can enhance osteoclast and adipocyte formation and cause bone loss, and that RES supplementation at 1 mg/kg may potentially prevent these bone defects.

## 1. Introduction

Chemotherapy is often the first line modality commonly used in the treatment of various types of cancer-including solid tumours and leukaemia [1,2]. Multiple chemotherapeutic agents with distinctly different modes of action are commonly used in cancer treatment [3]. However, one of the most challenging aspects of chemotherapy is the associated side effects in many organs, including severe long-term skeletal defects, which can negatively impact the quality of life of cancer patients and survivors. Experimental studies in rats have demonstrated that methotrexate (MTX) chemotherapy (which is commonly used in childhood oncology) significantly reduces growth plate thickness [4,5]. Additionally, MTX chemotherapy was found to suppress chondrocyte proliferation and to increase chondrocyte apoptosis [4]. Therefore, dysfunction of the growth plate induced by MTX chemotherapy is suggested to cause bone growth arrest. Moreover, previous studies in rats revealed a significant reduction in the volume of metaphysis trabecular bone (newly-formed bone derived from the growth plate), particularly at the secondary spongiosa and a significant increase in bone marrow fat content (adiposity) following MTX chemotherapy [4,5,6,7]. Furthermore, the direct damaging effects of MTX on osteoblasts, preosteoblasts, osteoprogenitors, bone marrow stromal progenitor cells, as well as osteocytes, are suggested to interfere with normal bone formation, bone marrow stromal progenitor cell osteogenesis/adipogenesis balance, and bone remodelling [4,6,7,8,9,10]. Additionally, osteoclast formation and activities were found to be aggravated following MTX chemotherapy [5,11,12]. Thus, a disturbance in the tightly controlled processes of bone formation, marrow fat formation, and bone resorption as a result of MTX chemotherapy has been thought to lead to bone growth defects.

Despite these previous studies, there is currently a lack of specific treatments for chemotherapy-induced bone defects and, thus, chemotherapy-associated adverse effects and preventive strategies have become a focus of research. While the current pharmacological anabolic or antiresorptive therapeutic approaches (e.g., using bisphosphonates, raloxifene, strontium ranelate, teriparatide, and denosumab) are being limited only to treat age-related osteoporosis [13], there is an acute need for novel, safe, and cost-effective strategies, not only to combat the skeletal toxicity related to MTX chemotherapy, but also for preserving bone mass and possibly restoring bone growth [14]. In recent years, attention has been focused on searching and using natural compounds and bioactive micronutrients as potential alternatives to preserve bone mass and bone growth during childhood cancer chemotherapy.

Resveratrol (RES) (3,4′,5-trihydroxy-*trans*-stilbene) is a naturally-occurring polyphenol produced by various plants, including red grapes, peanuts, mulberries, and berries [15]. Many previous studies have shown that RES is a potent anti-oxidant and anti-inflammatory agent and it provides protection against cancer, cardiovascular, and age-related diseases. In addition, many studies have been conducted to investigate its bone protective effects and action mechanisms. RES has been identified as a potent activator of sirtuin 1 (Sirt1), which is also known as NAD-dependent deacetylase [16]. The activation of Sirt1 has been shown to downregulate in vitro preadipocyte proliferation and adipogenic differentiation by inhibiting the transcription activity of adipogenic transcription factors PPARγ and C/EBPα [17,18,19,20]. RES has also been shown to augment Wnt signalling, thus enhancing osteoblast formation [21]. Moreover, the bone protective effects of RES have been demonstrated in a recent in vitro cell culture model by the inhibition of receptor activator of nuclear factor kappa-B (NF-κB) ligand (RANKL)-induced osteoclastogenesis which is independent of Sirt1 activation [22]. In addition, by masking the expression of proinflammatory cytokines (IL-1, IL-6 and TNF-α) and NF-κB signalling, the anti-inflammatory effects of RES is suggested to have a potential role in suppressing osteoclastogenesis and bone resorption [23,24,25]. Furthermore, several studies have also investigated the potential phytoestrogenic and, thus, bone protective effects of RES in ovariectomised or aging rats [26,27,28].

While the bone protective effects of RES seem promising, the potential of RES to prevent and restore MTX-related bone loss has not been explored. Our study aimed to gain more insights into the potential bone protective effects and action mechanisms of RES supplementation (1 mg/kg) in preventing MTX chemotherapy-induced bone defects using an acute MTX chemotherapy model in young rats.

## 2. Materials and Methods

### 2.1. Animal Trials and Specimen Collection

All procedures were approved by the Animal Ethics Committees of the University of South Australia and Institute of Medical and Veterinary Sciences (IMVS, Adelaide, South Australia, Australia). Two separate trials were conducted with six-week-old male Sprague-Dawley rats, which were randomly allocated into treatment groups: Control, RES, MTX, and MTX + RES-treated groups, respectively (*n* = 6). Rats were subcutaneously injected with MTX once daily for five days at 0.75 mg/kg, a dose similar to clinical therapeutic usage. Control rats were given only saline during the same period. RES (ResVida^®^, a *trans*-resveratrol from DSM (Basel, Switzerland) was orally gavaged once daily at 10 mg/kg (first trial) or 1 mg/kg (second trial) [29,30] in 0.5% methyl cellulose and aminoguanidine hemisulfate (Sigma, NSW, Australia). Rats were gavaged once daily for seven days prior to the first MTX injection and then once daily during the five-day MTX injections. The control group was administered with vehicle oral gavage only. All rats were humanely killed following CO_2_ overdose for collection of bone specimens on day 9 after initial MTX injection. Both the right and left tibias were dissected free from soft tissues. The left tibia specimens were fixed in 10% formalin for 24 h, decalcified in formic acid-based bone decalcifying solution, Immunocal (Decal Corp., Tallman, NY, USA) for seven days at 4 °C, processed, and embedded in paraffin wax. From the right tibia, metaphyseal sample was collected and stored at −80 °C until RNA isolation.

### 2.2. Ex Vivo Osteoclast Formation Potential of Bone Marrow Cells of Treated Rats

Bone marrow was flushed out from both femurs of treated rats with basal minimal essential medium (α-MEM) and cultured as described [12]. After an overnight culture, the non-adherent cells were collected and cultured at 1 × 10^6^ cells/well for one day in basal media containing 10 ng/mL M-CSF. Then, the basal media supplemented with 10 ng/mL M-CSF and 30 ng/mL RANKL was used the cells were cultured for seven days with media change every 2–3 days. After fixation in 10% formalin, osteoclasts were identified by tartarate-resistant acidic phosphotase (TRAP) staining, and TRAP+ multinuclear (≥3 nuclei) cells were counted as described [12].

### 2.3. Histomorphometric Analyses of Growth Plate and Metaphysis Bone

Paraffin sections of 4 μm thick were cut from the left tibia, de-waxed and stained with haematoxylin and eosin (H and E) as well as for tartrate-resistant acidic phosphatase (TRAP, a marker for osteoclasts) [9]. Stained sections were used for morphometric measurements of total heights [4,6] of the growth plate (a growth cartilage responsible for bone lengthening). In the metaphysis region, heights of the primary spongiosa (the mineralized cartilage trabeculae derived from the growth plate) and osteoblast density measurements were made, as described [4,7]. Briefly, primary spongiosa heights were obtained by measuring the heights between the end of growth plate and the top of secondary spongiosa. Osteoblast density was obtained by counting cuboidal mononuclear cells along the trabecular surface in six sequential images along primary spongiosa, and expressed as osteoblast number per mm^2^ trabecular bone area. TRAP+ multinuclear osteoclasts were counted under light microscopy and expressed as cells per mm^2^ trabecular bone area in the secondary spongiosa as described [31,32]. Adipocytes within bone marrow area were counted in four random images within the lower secondary spongiosa region, and expressed as adipocyte number per mm^2^ marrow area [7,10].

To determine treatment effects on bone architecture, trabecular number (number/mm), thickness (mm) and bone volume fraction (trabecular bone volume/total tissue volume, BV/TV%) were analysed within the secondary spongiosa at a position 1 mm below the primary and secondary spongiosa transitional line [4]. The stained sections were analysed by image analysis software analySIS^®^ Cell Imaging Analyzer (Olympus Soft Imaging Solutions, Münster, Germany) under a light microscope. The above measurements were made on three separate sections of 200 μm interval and averaged for each rat. 

### 2.4. Quantitative Real Time Reverse Transcription Polymerase Chain Reaction (RT-PCR)

Real time RT-PCR was performed to examine the treatment effects on expression of genes known important in regulating osteoblast, osteoclast and adipocyte formation. Briefly, frozen metaphyseal samples were crushed to a fine powder by mortar and pestle in liquid nitrogen, and RNA extraction was then performed with TRI reagent (Sigma). Two micrograms of total RNA from each sample was then reversed transcribed to single stranded cDNA by using a high-capacity RNA-to-cDNA kit (Applied Biosystems, Foster City, CA, USA).

All PCR primers were designed using rat DNA sequences (Table 1). RT-PCR was performed in a 10 μL reaction mixture containing cDNA from each sample, forward and reward primers of the gene of interest and SYBR^®^ Green master mix (Applied Biosystems). Quantitative PCR was performed using 7500 Fast Real-time PCR system (Applied Biosystems) in duplicate with the cycling conditions according to manufacturer’s protocol. PCR conditions were as followed: 60 °C for 30 s, 95 °C for 10 min, 40 cycles at 95 °C for 15 s, and 60 °C for 1.5 min, followed by a melt curve analysis. From the amplification curves, the relative gene expression was calculated using the comparative Ct (2-∆Ct) method, where threshold cycle (Ct) values from the gene of interest in duplicate runs were averaged and calibrated with the Ct values from the internal control, cyclophilin A [7].

### 2.5. Statistics

The data were analysed by a one-way analysis of variance (ANOVA) in GraphPad Instat (Version 3.0) (San Diego, CA, USA) and presented as means ± SEM. When the significance levels (*p* < 0.05) were achieved following ANOVA, a Tukey’s post hoc test was performed to identify the mean values which were significantly different with each other in the dataset. In the figures, the symbols *, **, and *** represent *p* < 0.05, *p* < 0.01, and *p* < 0.001, respectively.

## 3. Results

### 3.1. Effects of RES Supplementation at the Higher Dose

**Figure 1:**
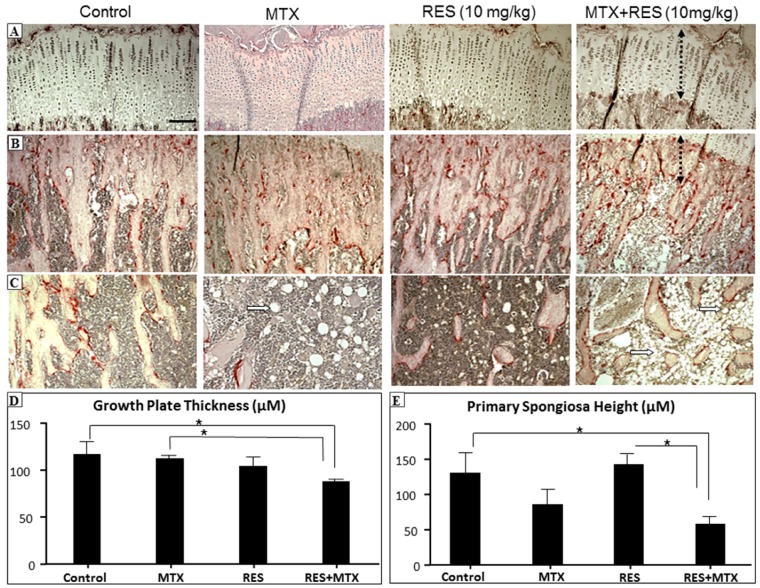
The effects of acute MTX treatment with or without resveratrol (RES) supplementation at 10 mg/kg dosage on growth plate and primary spongiosa heights and bone marrow fat content in the tibia of young rats. H and E and tartarate-resistant acidic phosphotase (TRAP) staining images showing effects of four different treatments on (**A**) growth plate thickness (bar = 50 μm, which applies to other images); (**B**) primary spongiosa heights (dashed lines); (**C**) bone marrow adipocytes (arrows) in the lower secondary spongiosa; (**D**) measurements of growth plate thickness; and (**E**) measurements of primary spongiosa heights. * *p* < 0.05.

After treatments of MTX, with or without RES, at the higher daily dose 10 mg/kg, effects on the bone were examined by histological analyses. While MTX treatment alone had a trend of causing reductions in growth plate thickness and metaphyseal primary spongiosa height when compared to control, RES + MTX combination treatment caused further reduction in the growth plate and primary spongiosa heights (Figure 1A,B,D,E, *p <* 0.05) when compared to the normal controls. However, RES alone at this dosage did not appear to affect these two measurements. Furthermore, RES + MTX combination treatment also further increased the fat content in the bone marrow (Figure 1C). These data suggest that combination usage of MTX with RES at 10 mg/kg dose further aggravate the MTX bone damage effect. Consequently, this study has focused analyses more on the supplementation treatment effect of RES at 1 mg/kg dose.

### 3.2. Effects of RES Supplementation (1 mg/kg) on Structures of Growth Plate and Metaphysis

**Figure 2:**
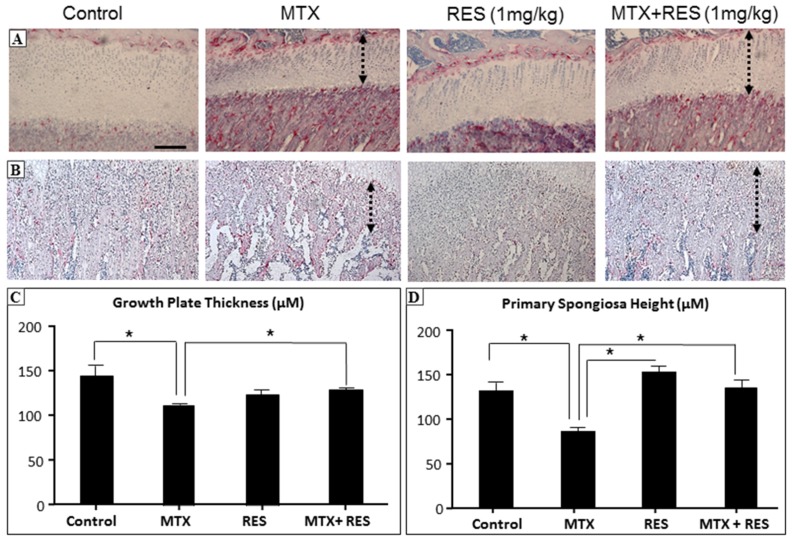
The effects of acute MTX treatment with or without resveratrol (RES) supplementation at 1 mg/kg dosage on growth plate and primary spongiosa heights in the tibia of young rats. H and E and tartarate-resistant acidic phosphotase (TRAP) staining images showing effects of four different treatments on (**A**) growth plate thickness (bar = 50 μm, which applies to other images) and (**B**) primary spongiosa heights (dashed lines); (**C**) Measurements of growth plate thickness; and (**D**) measurements of primary spongiosa heights. * *p* < 0.05.

After treatments of MTX, with or without RES, at the lower daily dose 1 mg/kg, histological analyses showed that MTX treatment alone significantly reduced growth plate height when compared to control (Figure 2A,C, *p <* 0.05). However, RES + MTX treatment preserved the growth plate height (Figure 2A,C, *p <* 0.05 compared to MTX alone group), while RES supplementation alone had no significant effects.

Metaphysis bone is made up with primary and secondary spongiosa, where the primary spongiosa consists of the mineralized cartilage derived from the growth plate, while secondary spongiosa is composed of enlarged mineralized bony trabeculae modelled or remodelled from the primary spongiosa. After acute MTX treatment at 0.75 mg/kg/day for five consecutive days, a significant reduction in primary spongiosa height was observed, which was prevented by the RES + MTX combination treatment (Figure 2B,D, *p <* 0.05).

**Figure 3:**
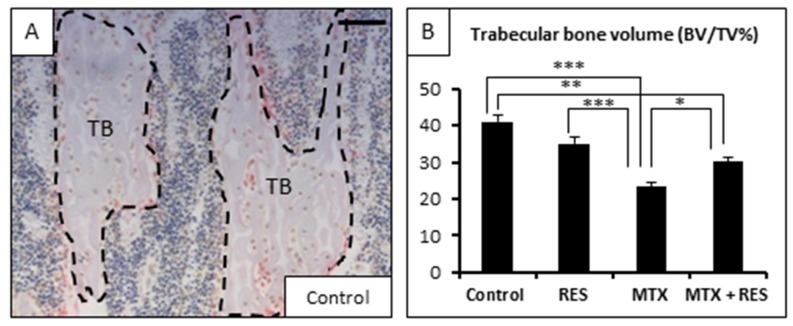

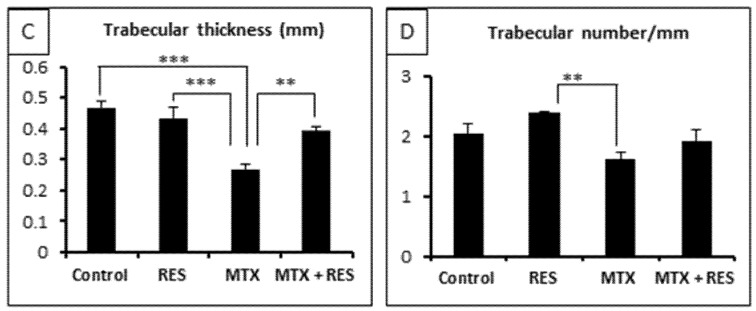
Effects of acute MTX treatment with and without resveratrol (RES) supplementation (1 mg/kg) on the trabecular bone volume and structure at the metaphysis secondary spongiosa of tibia of young rats. (**A**) A histological image showing traced trabecular bone (TB) of a control rat (bar = 125 μm); (**B**) effects on trabecular bone volume fraction BV/TV; (**C**) effects on trabecular thickness; and (**D**) effects on trabecular number. * *p* < 0.05, ** *p* < 0.01, *** *p* < 0.001.

Furthermore, trabecular bone volume in MTX alone-treated animals was significantly reduced compared to control (Figure 3A,B, *p <* 0.001). The reduction in bone volume fraction was associated with significant decrease in trabecular thickness (Figure 3C, *p <* 0.001 vs. normal control). The trabecular number was slightly lower compared to normal control (Figure 3D, *p* > 0.05). In comparison to the control group, RES treatment alone showed no significant changes in bone volume, trabecular thickness and number (Figure 3B–D). Interestingly, bone volume fractions in MTX + RES-treated rats were significantly higher compared to MTX alone-treated rats (*p <* 0.05), although they were still significantly lower than the normal controls (*p <* 0.01). In addition, the trabecular thickness in MTX + RES-treated rats was significantly greater than MTX alone-treated rats (*p <* 0.01) (Figure 3C) and the benefit effect of RES supplementation on trabecular number was slight in MTX + RES-treated rats (*p* > 0.05 vs. MTX alone-treated rats) (Figure 3D).

### 3.3. Treatment Effects on Osteoblast Number and Osteogenesis-Related Genes

**Figure 4:**
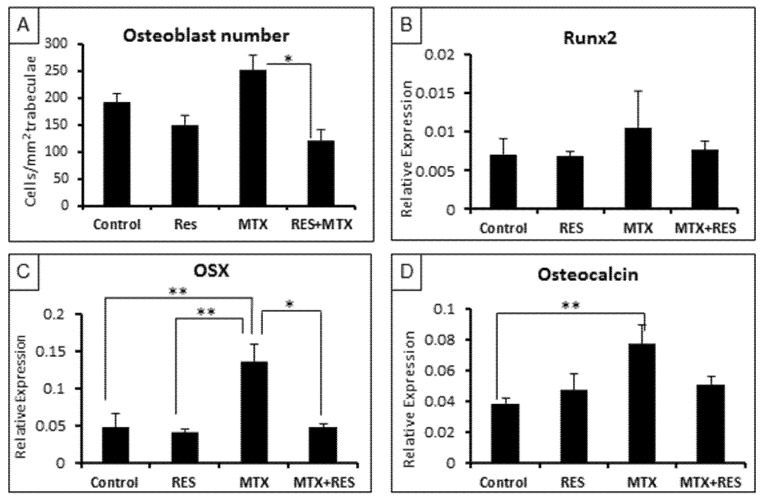
Effects of acute MTX treatment with and without resveratrol (RES) supplementation (1 mg/kg) on the osteoblast density and mRNA expression of osteogenesis-related genes at the metaphysis of tibia of young rats. (**A**) Osteoblast density on trabecular bone surface; (**B**) effects on expression of Runx2; (**C**) effects on expression of OSX; and (**D**) effects on expression of osteocalcin. * *p* < 0.05, ** *p* < 0.01.

To further examine treatment effects on osteogenesis, firstly, densities of osteoblasts on trabecular bone surfaces were measured. While RES alone had no significant effects on osteoblast density, MTX alone-treatment tended to increase it on day 9 following the first of five daily MTX treatments (*p >* 0.05 vs. normal control). However, MTX + RES combination treatment restored the osteoblast density (*p <* 0.05 vs. MTX alone) (Figure 4A).

Furthermore, real-time RT-PCR was carried out to access the treatment effects on the levels of expression of key osteogenesis-related genes. Consistent with the change pattern of the osteoblast density, levels of expression of osteogenic transcription factors (the earlier factor Runx2, and the later factor Osx) and the specific bone matrix protein osteocalcin showed similar patterns of changes upon various treatments (Figure 4B–D). While effects on Runx2 were very slight, expression levels of OSX and osteocalcin were significantly increased by MTX treatment alone (*p <* 0.01 vs. normal controls), but they were restored in the MTX + RES-treated rats (*p >* 0.05 vs. normal controls) (Figure 4B–D).

### 3.4. Changes in Adipocyte Density in the Bone Marrow

**Figure 5:**
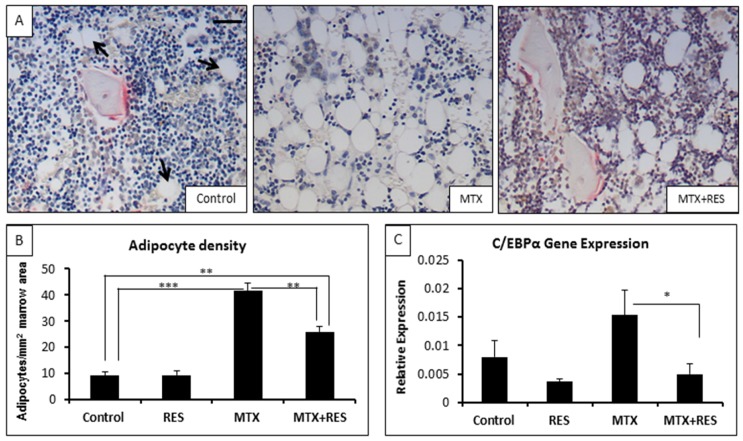
Effects of acute MTX treatment with and without resveratrol (RES) supplementation (1 mg/kg) on the adipocyte density at the bone marrow of metaphysis lower secondary spongiosa of tibia of young rats. (**A**) Representative histology images of various groups at the lower secondary spongiosa (bar = 25 μm); (**B**) effects on adipocyte cell density in the bone marrow; and (**C**) effects on mRNA expression of adipogenesis transcription factor c/EBPα. * *p* < 0.05, ** *p* < 0.01, *** *p* < 0.001.

To examine the treatment effects on bone marrow adipogenesis potential, adipocyte densities in the bone marrow at the lower secondary spongiosa in control, MTX alone-, RES alone-, and MTX + RES-treated rats were measured (Figure 5A,B). After the acute MTX chemotherapy, there was a dramatic increase in adipocyte number per mm^2^ bone marrow area (excluding trabecular area) compared to the control group (Figure 5A,B, *p <* 0.001). RES-alone-treated rats had no significant changes in adipocytes number compared with the control group. Supplementation with 1 mg/kg of RES in MTX-treated rats had a significant beneficial effect in reducing adipocyte density (Figure 5A,B, *p <* 0.01 vs. MTX-treated rats), although it did not completely suppress the adipogenesis (*p <* 0.01 vs. normal control). 

Albeit not significant, RT-PCR analysis of adipogenesis transcription factor gene C/EBPα revealed a trend of increased expression of C/EBPα following acute MTX chemotherapy (Figure 5C, *p >* 0.05 vs. control). However, following MTX + RES combination treatment, C/EBPα expression levels were restored to normal levels (Figure 5C, *p >* 0.05 vs. control; *p <* 0.01 vs. MTX alone).

### 3.5. Effects on Osteoclast Density and Osteoclast Formation

**Figure 6:**
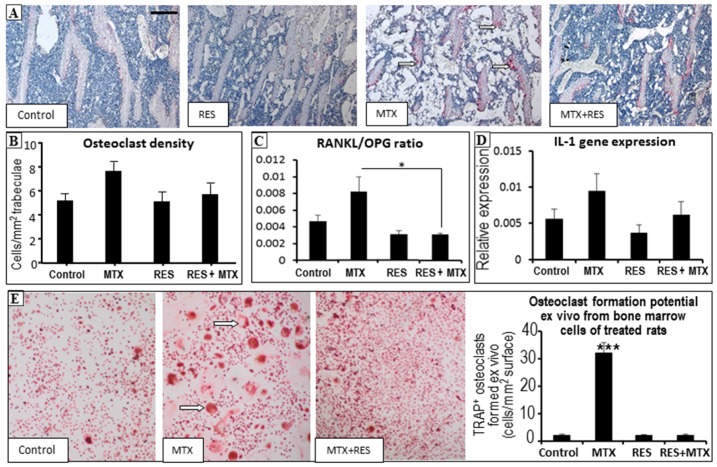
Effects of acute MTX treatment with and without resveratrol (RES) supplementation (1 mg/kg) on the osteoclast density and osteoclast formation potential in tibia of young rats. (**A**) Representative histology images (red colour TRAP staining as osteoclasts as indicated by arrows) of various groups at the secondary spongiosa (bar = 250 μm); (**B**) effects on osteoclast density on trabecular bone surface at the secondary spongiosa; (**C**) effects on mRNA expression ratio of osteoclastogenic factor RANKL and inhibitor OPG; (**D**) effects on mRNA expression of pro-osteoclastogenic cytokine IL-1; and (**E**) effects on osteoclast formation potentials (formed multinuclear TRAP-stained osteoclasts as indicated by arrows) of bone marrow cells from rats of various treatment groups. * *p* < 0.05, *** *p* < 0.001.

Finally, treatment effects on densities of bone-resorbing osteoclasts and osteoclast formation were examined. Albeit not significant, quantitative analyses of bone-resorbing osteoclasts on trabecular bone surfaces revealed a trend of increased osteoclast density following the acute MTX treatment (Figure 6A,B, *p >* 0.05 vs. control). However, following MTX + RES combination treatment, the osteoclast density was restored to the normal level (Figure 6B).

The impact of treatments on mRNA expression of osteoclastogenic factors was also examined in this study using RT-PCR. The expression of RANKL and OPG in the metaphyseal bone was shown to have a similar trend; however, MTX-treated rats had a trend of increase in the RANKL/OPG ratio when compared to the normal control rats (*p >* 0.05), but a significantly higher RANKL/OPG ratio when compared to MTX + RES-treated rats (*p <* 0.05) (Figure 6C). Moreover, gene expression patterns of the proinflammatory cytokines (TNF-α, IL-1, and IL-6) which promote osteoclastogenesis, were found to be in a similar trend of changes following different treatments albeit the lack of significant changes (Figure 6D).

Furthermore, the effect of RES treatment in vivo upon the ex vivo osteoclast formation potential of the bone marrow cells of treated rats was examined in M-CSF and RANKL-induced osteoclastogenesis assays. TRAP+ multinucleated (≥3 nuclei) osteoclasts formed were present in cultures from all treatment groups, but were abundant in a significantly greater number in the MTX-alone-treated group (*p <* 0.001 vs. all other groups) (Figure 6E). However, the combination treatment of MTX with RES significantly suppressed MTX-induced osteoclastogenesis (*p <* 0.001 vs. MTX-alone group) (Figure 6E).

## 4. Discussion

The rapid advances in the field of oncology with intensive chemotherapy result in improved cure rates, especially for paediatric acute lymphoblastic leukaemia patients [33]. However, one of the major health threats associated with these effective chemotherapy regimens is the irreversible detrimental effects on bone. Currently, therapeutic approaches to successfully prevent or control bone loss caused by MTX chemotherapy are still lacking. RES, a naturally-occurring molecule, has been identified to have moderate protective effects in optimising bone health including age-related bone loss despite the unclear mechanisms [30,34]. However, the potential bone protective effects of RES in the presence of MTX chemotherapy are largely unknown and remain to be fully elucidated. The current study demonstrated a significantly reduced growth plate thickness, shorter primary spongiosa height, reduced trabecular bone volume, as well as increased marrow adiposity following acute MTX treatment. However, RES supplementation demonstrated protective effects against MTX-induced bone damage in this rat model study.

A significant reduction in bone volume fraction was observed on day 9 after the initial MTX injection. The result is consistent with our previous findings in which the most severe trabecular structural damage was observed on days 9 to 11, which was restored to control levels on day 14 after the initial MTX treatment [4,6]. Interestingly, in the present study, RES supplementation at 1 mg/kg was found to be able to prevent the reduction in bone volume fraction in metaphyseal secondary spongiosa after acute MTX chemotherapy. Consistently, we have shown a significant reduction in the thickness of the growth plate and shorter primary spongiosa height after MTX chemotherapy compared to normal control, a damage which was prevented in RES-supplemented MTX-treated rats. Previously, the damaging effect on the trabecular structure after MTX chemotherapy has been suggested to be related to the interference in the production of calcified cartilage from the growth plate to form mature trabecular bone [4]. Encouragingly, these current findings found that RES was able to prevent the reduction in growth plate thickness and primary spongiosa height, as well as trabecular bone loss, after acute MTX chemotherapy. Therefore, RES can potentially prevent the negative effects of MTX on both the growth plate and metaphysis and, therefore, enables normal endochondral bone growth and bone mass deposition.

In addition, the observed damaging effects on trabecular structure and volume in the metaphysis caused by acute MTX chemotherapy could be attributed to an imbalance in the bone remodelling process with reduced bone formation and increased bone resorption. Our analyses have shown that MTX chemotherapy caused an increased osteoclast formation in the bone marrow, resulting in an elevated osteoclast density on the bone surface, and that RES supplementation could partially prevent these adverse effects. Consistently, our gene expression studies on the osteoclastogenesis signal (RANKL/OPG ratio) and proinflammatory cytokines (TNF-α, IL-1 and IL-6) revealed that the mRNA expression levels of these genes were upregulated in MTX-treated rats to different extents compared to control rats. RES supplementation appeared to be able to reduce the RANKL/OPG ratio and expression levels of IL-1. These findings suggest the important role of these osteoclastogenic factors in promoting osteoclast formation, subsequently resulting in excessive bone resorption following MTX chemotherapy. On the other hand, RES supplementary treatment during MTX chemotherapy caused a balanced increase in both RANKL and OPG transcription activities so that it has counteracted the effects of MTX chemotherapy and returned the RANKL/OPG ratio to control levels. Consistent with our finding, an in vitro study has demonstrated that resveratrol at 1, 3, and 10 μM inhibited RANKL-induced osteoclastogenesis and induced apoptosis of differentiated osteoclasts [22]. Importantly, these findings further suggest that RES is a potential candidate to restore the balance between bone formation and resorption, thereby recalibrating the bone remodelling to a new steady state following MTX chemotherapy.

RT-PCR gene expression analyses showed that the osteoblastogenic transcription factors (Runx2 and Osx), as well as the specific bone formation marker (osteocalcin) in the metaphysis were upregulated in MTX-treated rats compared to the control group. The results indicate that there is an increase in osteoblast activity on day 9 after the initial MTX injection. Consistently, a time course study on the effects of MTX in rats has shown that the mRNA expression of osteocalcin in the metaphysis was upregulated on day 9 after the initial MTX injection [4]. In addition, a more recent study examining the osteogenic differentiation potential of bone marrow stromal cells isolated from MTX-treated rats has revealed a reduction in the levels of Runx2 and Osx mRNA expression on day 6 followed by an increase on day 9 and day 14 after the initial MTX chemotherapy [7]. A possible explanation for the upregulation of these osteoblastogenesis-related factors is that a recovery mechanism occurred in which compensatory bone formation is induced to replenish the bone loss induced by MTX chemotherapy in these experimental settings [4,7]. However, the temporal increase in the osteoblast activity on the remaining trabeculae in the metaphysis was still insufficient to prevent bone loss. The extent of recovery to fully restore bone loss as well as the maintenance of bone mass is presently undefined and remains to be fully elucidated [35]. Interestingly, the current study found that RES supplementary treatment was able to maintain the levels of expression of these osteogenesis-related factors (Runx2, Osx, and osteocalcin) similar to control levels following MTX chemotherapy. RES possibly may have maintained bone homeostasis in the metaphysis by regulating the balance between bone resorption and bone formation and, thus, no overly elevated expression of osteogenesis factors has been seen in MTX + RES-treated rats in this study.

Furthermore, MTX was found not only to disturb the dynamics of bone homeostasis, but to alter bone marrow adipocyte population. The current study examining adipocyte density in the bone marrow at the secondary spongiosa, as well as at the diaphysis region (data not shown), revealed that the adipocyte number was significantly increased on day 9 following acute MTX chemotherapy. This result accords with an earlier finding from our lab which has demonstrated that MTX increases marrow adiposity in both short and long term of MTX chemotherapy models [7,36]. Consistently, gene expression analyses from the present study showed that the transcription activity of C/EBPα was slightly elevated after MTX chemotherapy. As osteoblasts and adipocytes share common precursor cells in the bone marrow, it is proposed that bone marrow stromal progenitor cells tend to differentiate in favour of adipocyte lineage over osteoblast lineage starting at an early time point (on day 6) following MTX chemotherapy, resulting in increased adipocyte formation [7]. This reciprocal relationship, therefore, results in reduced osteogenic potential, which is suggested to be insufficient to compensate the excessive osteoclastic activity during the recovery phase on day 9. Interestingly, RES supplementary treatment during acute MTX chemotherapy resulted in a significant reduction in adipocyte density. While the MTX treatment-induced osteogenesis/adipogenesis switch has been suggested to be related to an attenuation of the Wnt/β-catenin pathway [36], and several in vitro studies have proposed that the reduced adipogenic potential following RES supplementation is due to the activation of Sirt1 [17,24,37,38], the underlying mechanisms of the positive effects of RES in reducing fat cell formation and maintaining osteoblastogenesis in the metaphysis remain to be further studied.

Previously, oral consumption of trans-resveratrol at 20 mg/kg/day for 28 days did not adversely affect the health variables tested in healthy rats, suggesting that this high dose is not harmful in rats [29]. The current study observed that, while resveratrol supplementation for a short term at 10 mg/kg dosage did not affect bone health in normal rats, it worsened MTX-induced bone damage in the treated rats (including thinning of the growth plate and the newly-formed primary woven bone, depletion of bone marrow cells, and accumulation of bone marrow adipocytes). While further studies are required to understand the underlying mechanisms, these differential effects could be related to a possible drug interaction or synergistic effect on bone tissue with the combination treatment of resveratrol and MTX. This is also consistent with a recent study which showed a synergistic effect of resveratrol combined with radiation in killing the radioresistant prostate cancers in vitro and in enhancing radiation therapy of the mouse model of prostate cancer cell xenograft tumour [39]. Furthermore, our observations of the harmful effect at 10 mg/kg and yet the protective effect on bone at 1 mg/kg of resveratrol in this MTX chemotherapy setting support the view of the dose-dependency of resveratrol in its health benefits. Mukherjee et al. have proposed, after reviewing the literature, that, at a lower dose, resveratrol can act as an anti-apoptotic agent, providing cardioprotection and other health benefits, and that at a higher dose, however, resveratrol can act as a pro-apoptotic compound, inducing apoptosis in cancer cells and possibly normal tissue cells. Thus, resveratrol can be useful in maintaining health at a low dose; whereas at a high dose, it has pro-apoptotic actions not only on tumour cells, but on healthy cells [40]. Indeed, long-term low dose dietary resveratrol supplement was recently shown to reduce cardiovascular deterioration in chronic heart failure in rats [41], and the maximal improvement in the cerebrovascular function in type 2 diabetes mellitus patients was achieved only with the lowest dose of resveratrol used [42].

The current study has used a cancer-free treatment model in rats so to focus on studying MTX chemotherapy-induced bone defects. MTX is most commonly used in childhood cancer treatment, and its intensive high dose usage (with or without glucocorticoids) has been known to play the major role in bone loss in the patients and/or survivors of the major childhood cancer (acute lymphoblastic leukaemia or ALL) [43], and skeletal toxicity occurs regardless of the presence or types of ALL. However, future studies will be needed to investigate the potential treatment effects of resveratrol in modulating the adverse effects of MTX and other cancer drugs on bone and other tissues in cancer-bearing models, including ALL and other cancers [44,45]. 

Furthermore, while many previous cell models and animal preclinical studies have established that resveratrol can increase chemosensitivity to cancer cells and enhance cancer treatment, resveratrol can act as a promising anticancer agent [46,47,48], and is known to be rapidly metabolized, thus having poor oral bioavailability. While future studies are required to investigate any potential differential metabolism of resveratrol in animals, with or without cancer, it has been recently shown that resveratrol has a stronger anti-tumour ability than its metabolite resveratrol monosulfate, and that differential sensitivities of bladder cancer cell lines to resveratrol are not related to its metabolic profile [49].

## 5. Conclusions

In conclusion, we observed that, while RES supplementation for a short term at 10 mg/kg dosage did not affect bone health in normal rats, in MTX-treated rats it increased MTX-induced bone damage (for which the underlying mechanisms require further studies). However, we found that RES at 1 mg/kg dosage has the potential to confer protection against MTX chemotherapy-induced bone loss. Results obtained from this study have increased our understanding of RES’ effect in protecting bone during MTX treatment. It has been shown to have some partial rescue effect in preventing MTX chemotherapy-induced bone loss by preserving the growth plate structure and the endochondral bone growth function. Additionally, RES appeared to maintain the expression of osteogenic factors and was shown to reduce the extent of marrow adiposity induced by MTX chemotherapy. Furthermore, RES was shown to be able to suppress the expression of osteoclastogenic factors and inhibit osteoclast formation, suggesting its potential in preventing osteoclast formation and, thus, bone resorption following MTX chemotherapy. Therefore, RES is suggested to be a potential candidate for the development of preventative strategies to prevent the bone-related complications during and/or after MTX chemotherapy. Further studies are required to investigate its optimal dosage and its action mechanisms in protecting bone during cancer chemotherapy, including using cancer-bearing models.

## Figures and Tables

**Table 1:** Forward and reverse primer sequences used in the RT-PCR gene expression study.

Gene	Forward Primer (5′-3′)	Reverse Primer (5′-3′)
Cyclophilin A	GAGCTGTTTGCAGACAAAGTTC	CCTGGCACATGAATCCTGG
Runx2	TCACAAATCCTCCCCAAGTGG	GAATGCGCCCTTAAATCACTGA
OSX	GCTTTTCTGTGGCAAGAGGTTC	CTGATGTTTCTCAAGTGGTCG
Osteocalcin	AAGCCTTCATGTCCAAGCAGG	AGGCGGTGTTGAAGCCATACT
C/EBPα	TCGCCATGCCGGGAGAACTCTAAC	CTGGAGGTGGCTGCTCATCGGGG
RANKL	CCGTGCAAAGGGAATTACAAC	GAGCCACGAACCTTCCATCA
OPG	GGAGACACACCTCGCAAGA	CGACACTGCTTTCACAGAGG
TNF-α	ATGGCCCAGACCCTCACACTCAGA	CTCCGCTTGGTGGTTTGCTACGAC
IL-1	GTTTCCCTCCCTGCTCTGACA	GACAATGCTGCCTCGTGACC
IL-6	CAGCGATGATGCACTGTCAGA	CCAGGTAGAAACGGAACTCCA

Runx2: runt-related transcription factor 2; OSX: osterix; C/EBPα: CCAAT/enhancer-binding protein alpha; RANKL: receptor activator of nuclear factor kappa-B (NF-κB) ligand; OPG: osteoprotegerin; TNF: tumour necrosis factor; IL: interleukins.
